# Surface-Renewable AgNPs/CNT/rGO Nanocomposites as Bifunctional Impedimetric Sensors

**DOI:** 10.1007/s40820-016-0101-9

**Published:** 2016-09-13

**Authors:** Azadeh Azadbakht, Amir Reza Abbasi, Zohreh Derikvand, Ziba Karimi, Mahmoud Roushani

**Affiliations:** 1 Department of Chemistry, Faculty of Science, Khorramabad Branch, Islamic Azad University, Khorramabad, Iran; 2 Department of Chemistry, Ilam University, Ilam, Iran

**Keywords:** Silver nanoparticle, rGO, MoO_2_/Sal-His, Cysteine, Iodate

## Abstract

**Abstract:**

In this study, glassy carbon electrode modified by silver nanoparticles/carbon nanotube/reduced graphene oxide (AgNPs/CNT/rGO) composite has been utilized as a platform to immobilize cis-dioxomolybdenum (VI)–salicylaldehyde-histidine (MoO_2_/Sal-His). The modified electrode shows two reversible redox couples for MoO_2_/Sal-His. Electrocatalytic oxidation of cysteine (CySH) and electrocatalytic reduction of iodate on the surface of the modified electrode were investigated with cyclic voltammetry and electrochemical impedance spectroscopy methods. The presence of MoO_2_/Sal-His on AgNPs/CNT/rGO shifted the catalytic current of iodate reduction to a more positive potential and the catalytic current of cysteine oxidation to a more negative potential. The change of interfacial charge transfer resistance (*R*_ct_) recorded by the modified electrode was monitored for sensitive quantitative detection of CySH and iodate. Moreover, the sensor has a good stability, and it can be renewed easily and repeatedly through a mechanical or electrochemical process.

**Graphical Abstract:**

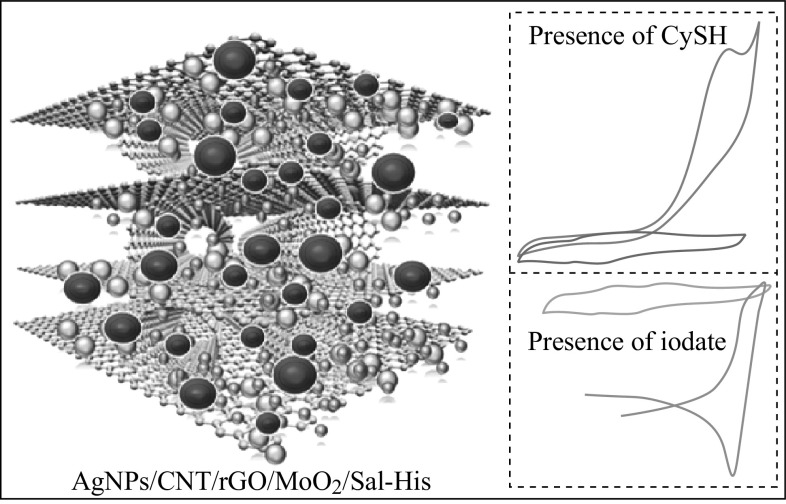

## Introduction

As a novel carbon material, graphene, with unique electrical and mechanical properties as well as large surface area, has received particular attention for potential applications in actuators, solar cells, field emission devices, field-effect transistors, supercapacitors, and batteries [1–3]. Due to van der Waals interactions, graphene nanosheets tend to agglomerate irreversibly to form graphite [4]. In order to obtain individual graphene sheets, graphene-based nanocomposites such as metal oxide/graphene, polymer/graphene, and nanoparticle/graphene have been successfully designed [5, 6]. Dispersion of metal nanoparticles on graphene sheets is an approach to reduce the aggregation, and therefore graphene-based nanocomposites containing nanoparticles were prepared and also to improve electronic and thermal conductivity. Nowadays, hybrid nanocomposites containing graphene sheets and metallic nanoparticles are expected to have promising potential applications in different areas such as chemical sensors, energy storage, catalysis, and hydrogen storage. Another important feature for attachment of metal nanoparticles to graphene is that the adhesion prohibits the aggregation of the graphene nanosheets in dry state. The metal nanoparticles also function as a spacer, therefore increasing the distance between the graphene nanosheets, which makes both faces of graphene accessible [7–9]. Recently, a simple synthetic route for high-density attachment of AgNPs onto the sides of graphene oxide (GO)–carbon nanotube (CNT) with high nanoparticle coverage has been reported by a simple one-step hydrothermal method without reducing agent [10].

l-Cysteine (CySH) due to its crucial role in biological systems has been the subject of many electrochemical studies from both mechanism and detection aspects [11]. Besides, it is widely used in the food industry as an antioxidant and its deficiency is associated with some disruptions such as slow growth, hair depigmentation, edema, lethargy, liver damage, muscle and fat loss, skin lesions, and weakness. As a consequence, its oxidation and accurate determination at low potential constitute a valuable task and the search for sensitive and selective methods for these purposes continues unabated. Various analytical methods have been applied for the determination of amino acids. Most of them are based on chromatographic separation [12], electrophoretic methods [13], and UV/Vis absorption spectrometry [14]. Among different methodologies used, electrochemical measurements of amino acids have gained considerable interest for electroactive compounds due to their simplicity, low cost, sensitivity, and feasibility for the development of in vivo sensors and chromatographic detectors. For this purpose, a variety of chemically modified electrodes (CMEs) with electrocatalytic properties have been employed in the detection of CySH [15, 16]. The reported CMEs have some limitations in linear dynamic range, selectivity, sensitivity, detection limit, and other characteristics. Consequently, it remains a great challenge to fabricate simple, low-cost, stable, sensitive, and selective CMEs that can improve the characteristics of electrocatalytic activity for determination of CySH. Furthermore, iodine is an essential micronutrient and plays a crucial role in the normal function of the thyroid gland. Iodine deficiency disorder (IDD) affects about two billion people and is the leading preventable cause of intellectual disabilities. As a result, detection of iodate (IO_3_^−^) is one of the important topics in food, clinical, and biological science [17].

Usually, an inorganic or organic electrocatalyst shows a single electroreductive or electrooxidative catalytic activity [18]. Designing a bifunctional electrocatalyst, especially those that catalyze both reductions and oxidations, has been a challenge in recent years [19, 20]. In this work, we used a simple and facile one-step method to prepare an AgNPs/CNT/rGO composite for immobilization of MoO_2_/Sal-His as a bifunctional sensor for the highly sensitive determination of CySH and iodate. The reduction of graphene oxide and the crystallization of the produced Ag were carried out concurrently in a single-step reaction by hydrothermal treatment. Silver nanoparticles with a small diameter were well distributed using an optimized amount of silver ammonia complex in the composite. Moreover, MoO_2_/Sal-His was immobilized on the GC electrode (the electrode denoted as AgNPs/CNT/rGO/MoO_2_/Sal-His) and then the modified electrode was utilized as a bifunctional sensor for determination of both CySH and iodate.

## Experimental

### Chemicals

Multiwalled carbon nanotubes with 95 % purity (10–20 nm diameter) and 1 µm length were obtained from Nanolab (Brighton, MA). Hydrochloric acid (37 %), potassium ferricyanide (K_3_Fe(CN)_6_), potassium ferrocyanide (K_4_Fe(CN)_6_·4H_2_O), potassium iodate, silver nitrate (AgNO_3_, 99.7 %), graphite powder (1–2 μm), potassium chloride (KCl), and CySH were purchased from Merck (Germany) and Fluka and were used as received without further purification. MoO_2_/Sal-His were synthesized, purified, and characterized as previously reported [21]. Solutions were deaerated by bubbling high-purity (99.99 %) nitrogen gas through them prior to the experiments. All experiments were carried out at the ambient temperature of 25 ± 1 °C. The electrochemical impedance spectroscopy (EIS) and cyclic voltammetry (CV) measurements were performed in the presence of 0.1 M PBS containing 0.1 M KCl and 3 mM [Fe(CN)6]^3−/4−^ or target molecules.

### Apparatus

Electrochemical experiments were performed using a μAutolab III potentiostat/galvanostat (Eco Chemie B.V.) with NOVA 1.8 software. A conventional three-electrode cell was used with an Ag/AgCl electrode (KCl 3 M) as the reference electrode, a Pt wire as the counter electrode, and a modified glassy carbon (GC) electrode as the working electrode. The cell was a one-compartment cell with an internal volume of 10 mL. JENWAY pH meter (model 3345) was used for pH measurements. To obtain information about morphology and size of the particles, scanning electron microscopy (SEM) was performed using an X-30 Philips instrument with an acceleration voltage of 25.0 kV. Also, Fourier transform infrared (FT-IR) and UV–Vis spectra were recorded by an AVATAR-370 Fourier transform infrared spectroscopy and a Unico 2800 UV/Vis spectrophotometer, respectively.

### Synthesis and Characterization of C_13_H_11_MoN_3_O_5_ or [MoO_2_ (Sal-His)]

The Mo (VI) complex was synthesized based on a previously reported procedure [21]. Firstly, 20 mL of salicylaldehyde solution (0.5 M) was slowly added into 20 mL histidine solution (0.5 M). Then 10 mL aqueous solution of sodium acetate (2 M) was added dropwise, the mixture was stirred for 1 h, and its color changed to deep yellow.

MoO_2_(acac)_2_ was also prepared according to a method reported in the literature [22]; then 10 mL of the prepared MoO_2_(acac)_2_ solution (0.85 M) was added to the above solution and the mixture was heated and refluxed for 5 h under vigorous stirring. The resulting yellow solid was filtered off, washed with several solvents, and dried at room temperature. The solid was dissolved in a minimum amount of DMF and the resulting solution was allowed to stand undisturbed. After a few days, yellow needle crystals were obtained by slow evaporation of the solvent at room temperature.

**Fig. 1:**
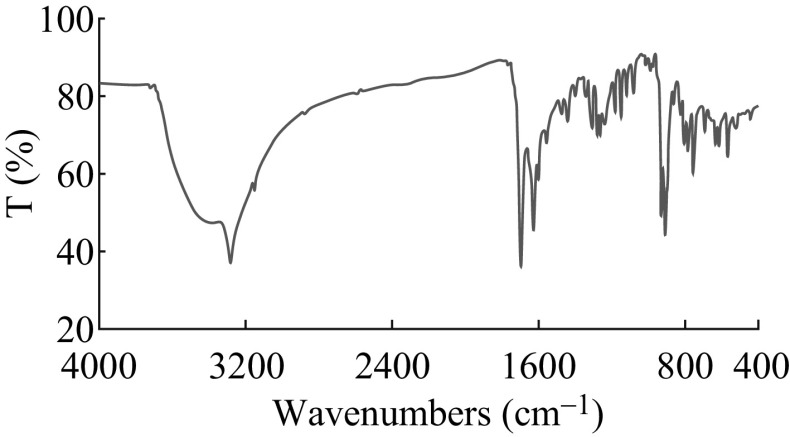
IR spectrum of MoO_2_ (Sal-His) complex

The IR spectrum of Mo (VI) complex shows a broad band in the 3274–3152 cm^−1^ region due to the stretching vibrations of physisorbed water molecules (Fig. 1). No band appeared near 1700 cm^−1^, confirming the complete deprotonation of carboxyl groups. The strong peaks at 1604 and 1350 cm^−1^ correspond to the asymmetric and symmetric stretching of carboxyl groups, respectively. The value of *Δ*[*v*_as_ _−_ *v*_s_] was 254 cm^−1^, comparatively larger than 200 cm^−1^, which is in agreement with the monodentate coordination of the carboxylate group [23]. The sharp peaks at 1556 and 1632 cm^−1^ are attributed to *v* (C=C) and *v* (C=N) of the aromatic rings, respectively. The bands in the 930–916 cm^−1^ region are due to Mo=O stretching vibrations [24]. Also, the bands in the range of 524 and 444 cm^−1^ are attributed to the Mo–O and Mo–N, generated by Mo complexation to the phenol O and azomethine N atoms, respectively [25].

**Fig. 2:**
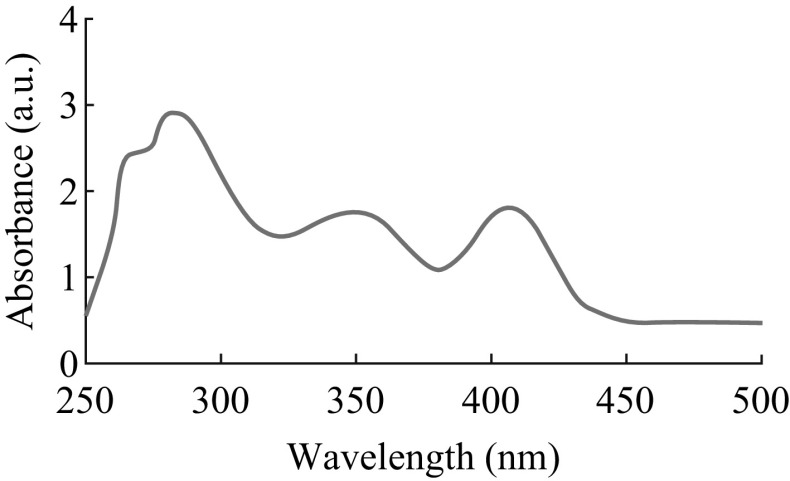
UV–Vis spectrum of MoO_2_ (Sal-His) complex

The electronic spectrum of the Mo (VI) complex exhibits absorption bands at 268, 280, and 350 nm which can be assigned to *π* → π* and *n* → π* intraligand transitions of benzene and imine (Fig. 2). The band appearing at 410 nm may be assigned to ligand-to-metal charge transfer (LMCT). This band was in the range usually observed for MoO complexes [26].

### Preparation of AgNPs/CNT/rGO Composite

In this study, graphene oxides were prepared from graphite via modified Hummers method [27]. For the synthesis of AgNPs/CNT/rGO, silver ammonia [Ag(NH_3_)_2_OH] solution was obtained by adding ammonia (1 wt %) to silver nitrate solution (50 mM) until the precipitates were visually invisible to the naked eye. The concentration of the synthesized Ag(NH_3_)_2_OH was approximately 40 mM. The water dispersions of CNT and GO (1.0 mg mL^−1^) were prepared in separate containers. The solutions of CNT and GO were mixed in the ratio of 3:1 (v/v). AgNPs/CNT/rGO was prepared by mixing the freshly prepared Ag(NH_3_)_2_OH solution with an aqueous solution of CNT/GO (1.0 mg mL^−1^) (3:1, v/v) in a volume ratio of 1:12 [10] under stirring for 30 min. The mixture was placed in an autoclave and heated at 170 °C for 4 h. After being cooled to room temperature, the product was isolated by centrifugation and the obtained precipitate was filtered and dried overnight at 50 °C.

### Electrode Modification

To prepare a modified electrode, GC electrode was polished with an emery paper followed by alumina (1.0 and 0.05 µm) and then thoroughly washed with double-distilled water. Afterward, this electrode was placed in an ethanol container and then a bath ultrasonic cleaner was used to remove the adsorbed particles. To obtain AgNPs/CNT/rGO nanocomposite-modified electrodes, 1 mg of AgNPs/CNT/rGO composite was dispersed in double-distilled water (1 mL) by 30-min mild ultrasonication to obtain a homogeneous suspension. After that, a certain amount (10 µL) of the prepared suspension was dropped onto the well-polished GC electrode surface and dried at room temperature for 24 h.

Afterward, the electrode was thoroughly rinsed with water and kept at room temperature for further use. For MoO_2_/Sal-His immobilization, MoO_2_/Sal-His was immobilized on the surface of the modified electrode by immersing AgNPs/CNT/rGO in 0.1 M phosphate buffer solution (PBS, pH = 2) containing 7 × 10^−3^ M MoO_2_/Sal-His. Then the electrode potential was cycled between −0.3 and 0.8 V at a scan rate of 50 mV s^−1^ for 40 cycles. The other used electrodes such as CNT/GC and rGO/GC electrodes were fabricated by casting 10 µL of CNT and rGO on the surface of bare GC electrode, respectively. In order to fabricate the AgNPs/GC electrode, AgNPs were electrochemically deposited by potentiostatic deposition, according to a one-step procedure reported in the literature [28], applying a constant potential of −0.40 V to a working electrode soaked in a solution containing 5 × 10^−3^ M AgNO_3_ and 0.1 M KNO_3_ for 10 s.

## Results and Discussion

### Characterization of the Modified Electrode by SEM

**Fig. 3:**
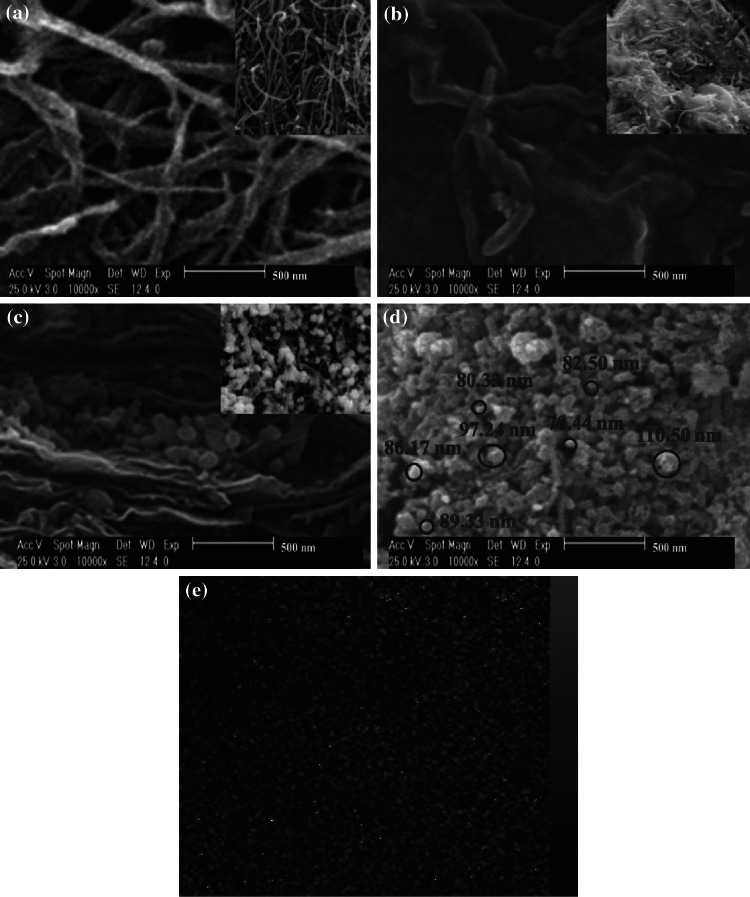
**a** Typical SEM images of CNT/GC, **b** CNT/rGO/GC, **c** AgNPs/CNT/rGO/GC, and **d** AgNPs/CNT/rGO/MoO_2_/Sal-His/GC electrodes; **e** EDX mapping analysis of AgNPs

Figure 3 shows the SEM images of CNT/GC (Fig. 3a), CNT/rGO/GC (Fig. 3b) AgNPs/CNT/rGO/GC (Fig. 3c), and AgNPs/CNT/rGO/MoO_2_/Sal-His/GC electrodes (Fig. 3d). As shown in Fig. 3b, it can be observed that the carbon nanotubes are impregnated in the matrix of rGO. As shown in Fig. 3c, the AgNPs are decorated on the crumpled thin layer of rGO and CNT. It can be seen in Fig. 3d that after MoO_2_/Sal-His immobilization, the surface of the AgNPs/CNT/rGO nanocomposite becomes rougher and the morphology of the electrode surface changed obviously, which implies that the MoO_2_/Sal-His is successfully deposited on the surface of the AgNPs/CNT/rGO electrode. This result indicates that the film has a globular structure with relatively homogeneous distribution in the range of 70–95 nm. To confirm the formation of AgNPs on the surface of electrode, energy-dispersive X-ray (EDX) mapping analysis was used, and the distribution of AgNPs is shown in Fig. 3e.

### Electrochemical Behavior of AgNPs/CNT/rGO

**Fig. 4:**
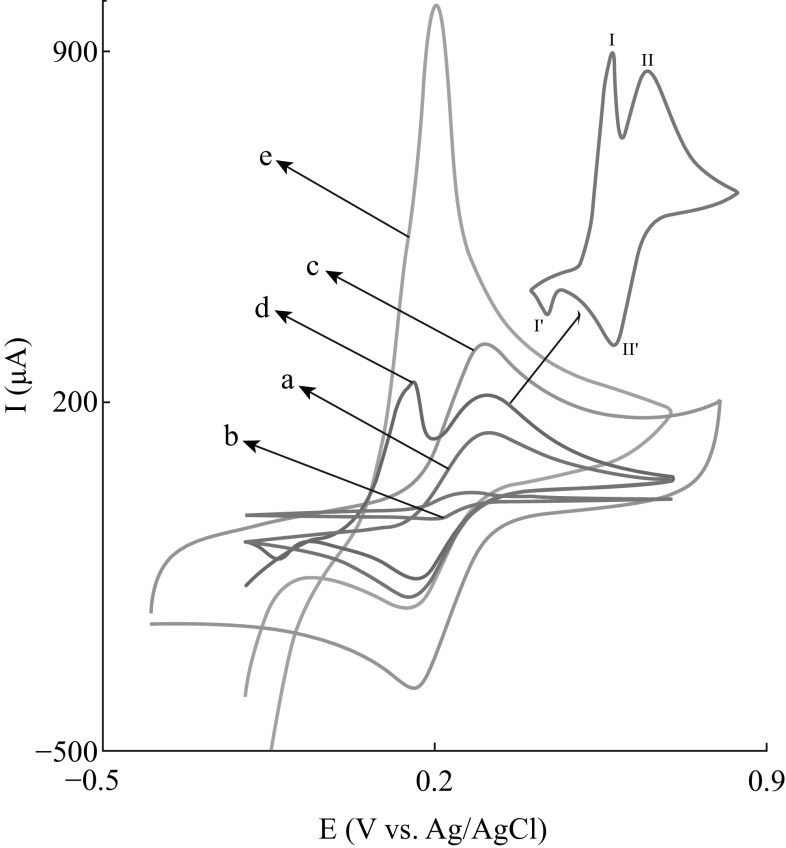
CV curves of *a* bare GC, *b* rGO/GC, *c* CNT/GC, *d* AgNPs/GC, and *e* AgNPs/CNT/rGO/GC in 0.1 M PBS (pH 6.0) containing 0.1 M KCl and 3 mM [Fe(CN)_6_]^3−/4−^ at a scan rate of 50 mV s^−1^

CV results of bare GC, rGO/GC, CNT/GC, AgNPs/GC, and AgNPs/CNT/rGO/GC in 0.1 M PBS (pH 6.0) containing 0.1 M KCl and 3 mM [Fe(CN)_6_]^3−/4−^ are shown in Fig. 4a–e. The CV curves of ferricyanide were chosen as a marker to investigate the changes of the electrode behavior before and after each assembly step. As it is obvious from Fig. 4, the CV curves of [Fe(CN)_6_]^3−/4−^ redox couple system at the bare GC, rGO/GC, and CNT/GC show a reversible one-electron redox wave. The peak currents of modified electrodes are considerably increased in the order of rGO/GC < bare GC < CNT/GC. In fact, CNT provides a large effective surface area of increased conductivity, compared with the cases of rGO and bare GC electrodes.

AgNPs/GC electrode (curve “*d*”) shows a very sharp oxidation peak at +0.15 V, followed by a single reduction feature at −0.1 V (redox peaks I–I’), confirming the successful immobilization of AgNPs on the GC electrode surface. Redox peaks II–II′ correspond to the [Fe(CN)_6_]^3−/4−^ redox couple. In order to enhance the surface area of the electrode, improvement of the electrode was carried out by casting AgNPs/CNT/rGO nanocomposite at the surface of GC electrode. As shown in curve “*e*”, the peak currents increase 2.5 times higher than that obtained with the CNT/GC electrode. Since the anodic peaks of both Ag/Ag^+^ and [Fe(CN)_6_]^3−/4−^ appear in the close potential region, the CV curves of AgNPs/CNT/rGO/GC containing [Fe(CN)_6_]^3−/4−^ is the sum of the electrochemical signals of Ag/Ag^+^ and [Fe(CN)_6_]^3−/4−^. These results clearly indicate that AgNPs/CNT/rGO has been successfully immobilized on GC electrode.

**Fig. 5:**
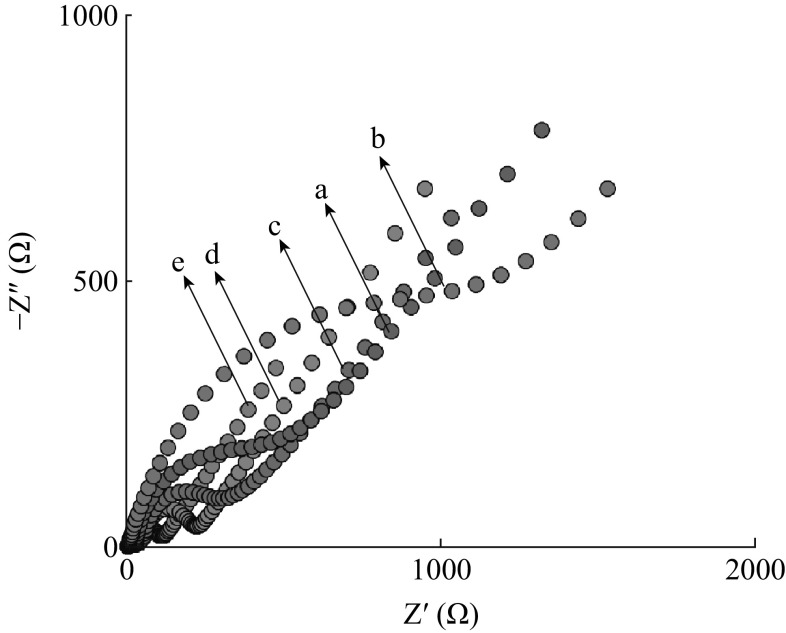
Nyquist plots for various electrodes of *a* GC, *b* rGO/GC, *c* AgNPs/GC, *d* CNT/GC, and *e* AgNPs/CNT/rGO/GC in 0.1 M PBS containing 0.1 M KCl and 3 mM Fe(CN)_6_^3−/4−^ in the frequency range of 10 kHz–0.1 Hz

Electrochemical impedance spectroscopy (EIS) is an effective technique for probing the features of surface-modified electrodes. Figure 5a–e, respectively, shows the typical Nyquist plots of GC, rGO/GC, AgNPs/GC, CNT/GC, and AgNPs/CNT/rGO/GC electrodes recorded in 0.1 M PBS solution containing 0.1 M KCl and 3 mM Fe(CN)_6_^3−/4−^ as an electrochemical redox marker. The straight line at low frequency is related to the diffusion process known as Warburg element, while the high-frequency semicircle is related to the electron transfer resistance (*R*_ct_), which controls the electron transfer kinetics of the redox probe at the electrode interface. The bare GC electrode shows a small electron transfer resistance indicating a fast electron transfer process (curve “*a*”). The diffusion-limited behavior at low frequencies is described by Warburg impedance. Modification of GC electrode with rGO effectively retarded the interfacial electron transfer kinetics of [Fe(CN)_6_]^3−/4−^ anions, which is reflected by the increase of *R*_ct_ value to about 1.2 kΩ (curve “*b*”). It indicates the hindrance to electron transfer, confirming the successful immobilization of rGO on GC electrode surface. Electrodeposition of AgNPs on the surface of GC electrode (curve “*c*”) results in the decrease of R_ct_ value compared with bare GC electrode. When CNT was casted on the surface of GC electrode, the value of *R*_ct_ was further decreased to about 270 Ω (curve “*d*”) compared with bare GC and AgNPs/GC electrodes, revealing the promoted conductivity of the CNT. Immobilization of AgNPs/CNT/rGO on the surface of the modified electrode causes a large decrease of *R*_ct_ value (curve “*e*”), indicating that the AgNPs/CNT/rGO film could provide good electron conduction pathways between the electrode and the electrolyte.

### Electrochemical Behavior of AgNPs/CNT/rGO/MoO_2_/Sal-His Electrode

CV curves of AgNPs/CNT/rGO/MoO_2_/Sal-His modified electrode were recorded in a pH 2.0 buffer solution. Immobilization of the MoO_2_/Sal-His on the AgNPs/CNT/rGO electrode shows two well-defined redox couples with the formal potentials (*E* ^0^′) of 0.22 and 0.41 V, respectively. The electrochemical reactions of two redox couples in acidic solutions might be given as follows [29]:

1 $${\text{Mo}}^{\text{VI}} {\text{O}}_{2} /{\text{Sal-His }} + 2{\text{H}}^{ + } + \, 2{\text{e}}^{ - } \to {\text{Mo}}^{\text{V}} {\text{O}}_{2} \left( {{\text{H}}_{2} {\text{O}}} \right)/{\text{Sal-His}}$$

2 $${\text{Mo}}^{\text{V}} {\text{O}}_{2} \left( {{\text{H}}_{2} {\text{O}}} \right)/{\text{Sal-His }} + 2{\text{H}}^{ + } + \, 2e^{ - } \to {\text{Mo}}^{\text{III}}_{{}} \left( {{\text{H}}_{2} {\text{O}}} \right)_{2} /{\text{Sal-His}}.$$

**Fig. 6:**
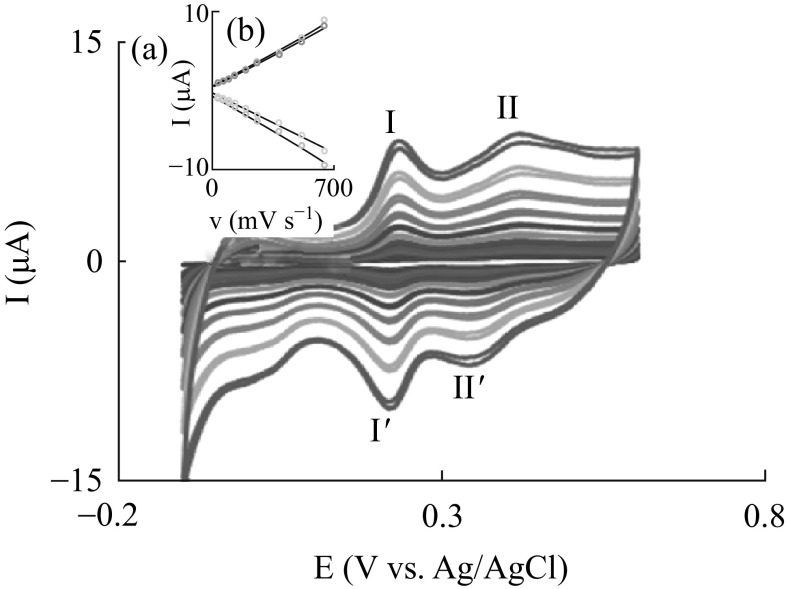
*a* CV curves of the AgNPs/CNT/rGO/MoO_2_/Sal-His electrode in PBS (pH 2.0) at different scan rates: 10, 25, 50, 75, 100, 150, 200, 300, 400, and 500 mV s^−1^. *b* Variation of the anodic and cathodic peak currents of the electrode versus potential scan rate

The CV curves of the AgNPs/CNT/rGO/MoO_2_/Sal-His modified electrode in PBS (pH 2.0) at different scan rates are shown in Fig. 6a. The peak currents of the voltammograms are linearly proportional to the scan rate (*ν*) of up to 200 mV s^−1^ for the peak currents of I–I and II–II′ (Fig. 6b), confirming the surface-type reactions. However, the peak potential differences (Δ*E*_p_) are larger than the theoretical value (0 mV) expected for a reversible surface redox process [30] and increase with the increasing scan rate, which might be due to the non-ideal behavior [31, 32].

### Electrocatalytic Activity of Modified Electrodes

**Fig. 7:**
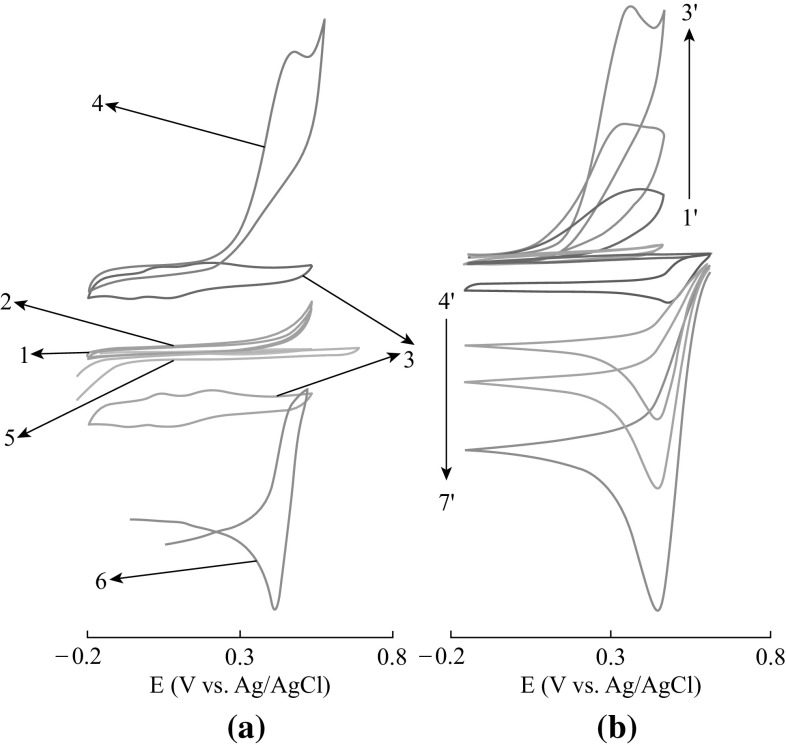
**a** CV curves of AgNPs/CNT/rGO/GC (1 and 2) and AgNPs/CNT/rGO/MoO_2_/Sal-His/GC (3 and 4) in the absence (1 and 3) and the presence (2 and 4) of 5 mM of CySH in 0.1 M PBS (pH 6.0) at a scan rate of 50 mV s^−1^. CV curves of AgNPs/CNT/rGO/GC (1 and 5) and AgNPs/CNT/rGO/MoO_2_/Sal-His/GC (3 and 6) in the absence (1 and 3) and the presence (5 and 6) of 2 mM iodate in 0.1 M PBS (pH 2.0) at a scan rate of 50 mV s^−1^. **b** CV curves of the AgNPs/CNT/rGO/MoO_2_/Sal-His/GC at a scan rate of 50 mV s^−1^ at different concentrations of CySH (1′–3′) of 5, 15, and 30 mM and iodate (4′–6′) of 0.5, 2, and 5 mM

Oxidation of CySH on the surface of AgNPs/CNT/rGO electrode and AgNPs/CNT/rGO/MoO_2_/Sal-His electrodes was investigated in 0.1 M PBS (pH 6.0). Figure 7a shows the recorded CV curves in the absence and the presence of 5 mM CySH in AgNPs/CNT/rGO (curves 1 and 2) and AgNPs/CNT/rGO/MoO_2_/Sal-His (curve 3 and 4) electrodes at a scan rate of 50 mV s^−1^. No electrochemical response was observed on AgNPs/CNT/rGO electrode in the absence of CySH (curve 1), but in the presence of CySH a small redox response can be observed at the potential of 0.6 V (curve 2). However, under the same conditions in AgNPs/CNT/rGO/MoO_2_/Sal-His electrode, the oxidation current of CySH starts at 0 V and an obvious catalytic oxidation occurs at the potential of 0.05 V (curve 4). It can be seen that the peak potential for oxidation of CySH is significantly shifted to a more negative potential (0.05 V vs. Ag/AgCl) compared with AgNPs/CNT/rGO modified electrode (0.6 V vs. Ag/AgCl). Furthermore, the anodic peak current is greatly enhanced in the presence of CySH and the reduction peak current totally disappeared, suggesting a typical electrocatalytic oxidation process. The substantially decreasing overvoltage and increasing oxidation peak current of CySH confirm that MoO_2_/Sal-His immobilized on AgNPs/CNT/rGO electrode can act as an efficient mediator to shuttle electrons between CySH and the working electrode. With increasing the concentration of CySH, the anodic current density is enhanced and the corresponding reduction current density is decreased (Fig. 7b, curve 1′–3′).

Electrocatalytic reduction of iodate on the surface of the proposed modified electrode was also evaluated by CV. Reduction peak current of iodate at the surface of AgNPs/CNT/rGO and AgNPs/CNT/rGO/MoO_2_/Sal-His electrodes was recorded in PBS (pH 2.0) containing 2 mM of iodate at a scan rate of 50 mV s^−1^. The reduction current of iodate appeared at the potential of −0.3 V at the surface of AgNPs/CNT/rGO in the PBS (pH 2.0) (curve 5). However, the presence of MoO_2_/Sal-His film on AgNPs/CNT/rGO shifts the catalytic current of iodate reduction to a more positive potential (curve 6) compared with AgNPs/CNT/rGO modified electrode. These results indicate that MoO_2_/Sal-His exhibits an excellent catalytic activity toward iodate reduction. With increasing the concentration of iodate, the cathodic current density is enhanced and the corresponding oxidation current density is decreased (Fig. 7b, curve 4′–7′).

**Fig. 8:**
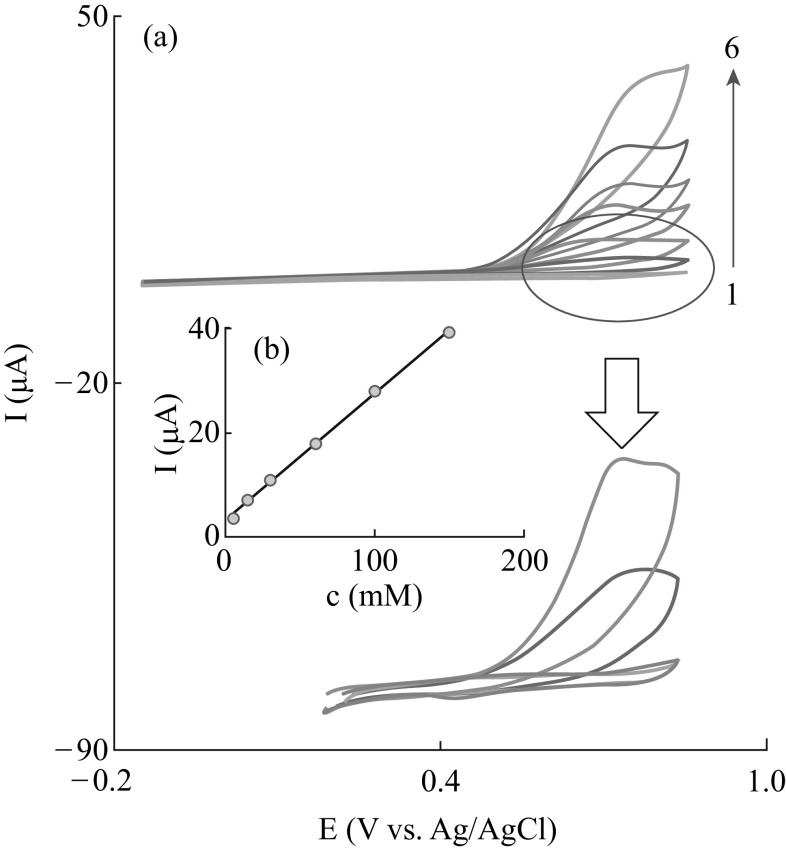
*a* CV curves of the AgNPs/CNT/rGO/MoO_2_/Sal-His/GC in the presence of CySH with different concentrations (1–6) of 5, 15, 30, 60,100, 150 mM in 0.1 M PBS (pH 6.0) at a scan rate of 50 mV s^−1^. *b* Plot of catalytic peak versus CySH concentration

Furthermore, CV curves of different concentrations of CySH at the modified electrode in 0.1 M PBS (pH 6.0) were recorded (Fig. 8a). The data reveal that the calibration curve is linear, with the CySH concentration in the range of 5–150 mM and a correlation coefficient of 0.996 (Fig. 8b).

As illustrated in Fig. 8a (magnification in Fig. 8a), when the concentration of CySH is enhanced, the anodic peak current of the modified electrode increases, whereas its cathodic peak current diminishes, indicating a typical electrocatalytic oxidation process (EC′). Hence, a possible mechanism of CySH electrooxidation on AgNPs/CNT/rGO/MoO_2_/Sal-His electrode might be given as follows:

3 $${\text{CySH}} \to {\text{Cys}}^{ - } + {\text{ H}}^{ + }$$

4 $${\text{Mo}}^{\text{III}}_{{}} \left( {{\text{H}}_{2} {\text{O}}} \right)_{2} /{\text{Sal-His}} \to {\text{Mo}}^{\text{V}} {\text{O}}_{2} \left( {{\text{H}}_{2} {\text{O}}} \right)/{\text{Sal-His }} + 2{\text{H}}^{ + } + \, 2{\text{e}}^{ - }$$

5 $${\text{Mo}}^{\text{V}} {\text{O}}_{2} \left( {{\text{H}}_{2} {\text{O}}} \right)/{\text{Sal-His }} + {\text{ Cys}}^{ - } + 2{\text{H}}^{ + } + {\text{e}}^{ - } \to {\text{Mo}}^{\text{III}}_{{}} \left( {{\text{H}}_{2} {\text{O}}} \right)_{2} /{\text{Sal-His }} + {\text{ Cys}}^{ \cdot }$$

6 $$2\;{\text{Cys}}^{ \cdot } \to {\text{CySSCy}}.$$

This is similar to the mechanism reported previously for CySH oxidation on solid electrodes [33, 34]. A possible mechanism for CySH oxidation at the surface of AgNPs/CNT/rGO/MoO_2_/Sal-His might be given as follows:

7 $${\text{CySH}} \leftrightarrow {\text{CyS}}^{ - }$$

8 $${\text{Cys}}^{ - } \to {\text{CyS}}^{ \cdot } + {\text{ e}}^{ - }$$

9 $$2{\text{CyS}}^{ - } \to {\text{CySSCy}}.$$

**Fig. 9:**
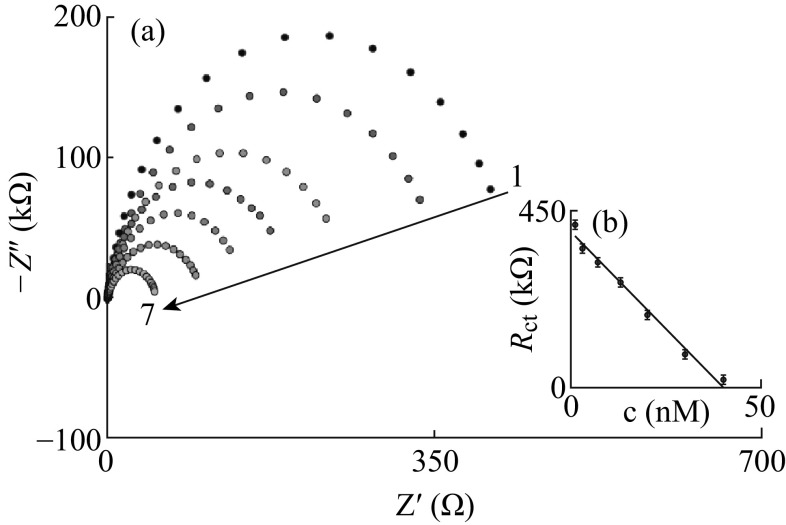
*a* EIS response of AgNPs/CNT/rGO/MoO_2_/Sal-His/GC in the presence of CySH with different concentrations (1–7) of 1, 3, 7, 13, 20, 30, and 40 nM in 0.1 M PBS (pH 6.0) at the frequency range of 10 kHz–0.1 Hz. *b* Corresponding calibration plot of *R*_ct_ versus CySH concentration

The EIS results obtained in the presence of increasing concentration of CySH at the surface of the modified AgNPs/CNT/rGO/MoO_2_/Sal-His electrode are shown in Fig. 9a. As shown in Fig. 9b, there is a linear relationship between *R*_ct_ and the concentration of CySH over a concentration range of 1.0 to 40.0 nM (*R* ^2^ = 0.992). The limit of detection (LOD) of this method was calculated at a concentration level of 300 pM.

**Fig. 10:**
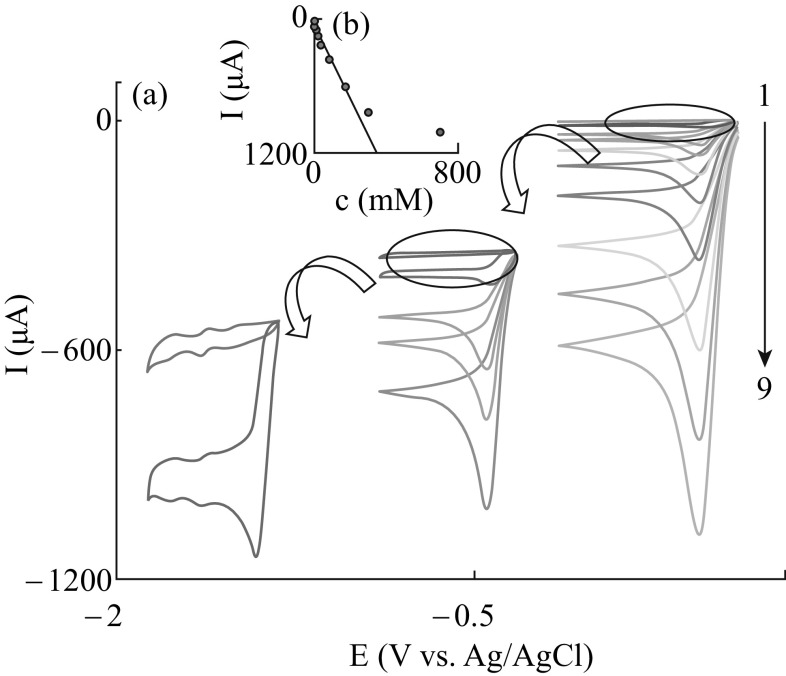
*a* CV curves of AgNPs/CNT/rGO/MoO_2_/Sal-His/GC in the presence of iodate with different concentrations (1–9) of 0.5, 2, 5, 15, 35, 80, 170, 300, and 700 mM in 0.1 M PBS (pH 2.0) at a scan rate of 50 mV s^−1^. *b* Plot of catalytic peak versus iodate concentration

Figure 10a shows the modified electrode response in the presence of different concentrations of iodate in 0.1 M PBS (pH 2.0). The cathodic peak current increases with increasing iodate concentrations in the range of 500 µM to 170 mM with a correlation coefficient of 0.996. As shown in Fig. 10b, the cathodic peak current is significantly enhanced in the presence of iodate accompanied by the disappearance of the oxidation peak, suggesting an excellent electrocatalytic reduction process. Increasing the concentration of iodate will increase the cathodic and reduce the anodic peak currents, indicating a typical electrocatalytic reduction process (EC′).

In order to optimize the electrocatalytic response condition, the effect of pH on the electrocatalytic activity of the modified electrodes for the reduction of iodate was examined by monitoring the CV curves of AgNPs/CNT/rGO/MoO_2_/Sal-His electrode in the presence of 2 mM of iodate in 0.1 M buffer solution with different pH values (not shown). In the pH range of 1–3, the modified electrode exhibits electrocatalytic activity, but the current density for iodate reduction decreases as the pH of solution increases from 3. The higher peak currents were observed at pH  2 because the reduction reaction of iodate depends strongly on the concentration of proton, as can be seen in Eq. (10):

10 $${\text{IO}}_{3}^{ - } + \, 6{\text{H}}^{ + } + \, 6{\text{e}}^{ - } \to {\text{I}}^{ - } + \, 3{\text{H}}_{2} {\text{O}}.$$

Impedimetric detection of iodate at the modified electrode under the optimized conditions was recorded and the relationship between *R*_ct_ values and the concentrations of iodate was studied.

**Fig. 11:**
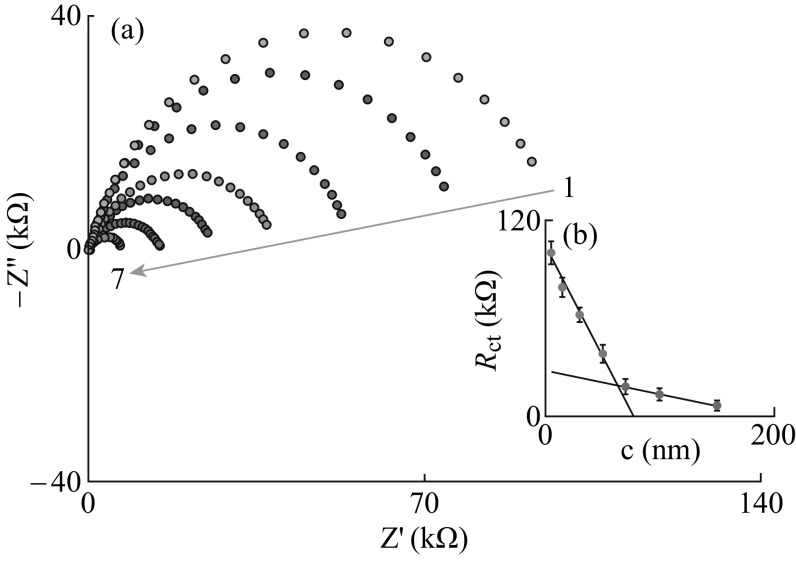
*a* EIS response of AgNPs/CNT/rGO/MoO_2_/Sal-His/GC in the presence of iodate with different concentrations (1–7) of 5, 15, 30, 50, 70, 100, and 150 nM in 0.1 M PBS (pH 2.0) in the frequency range of 10 kHz–0.1 Hz. *b* Corresponding calibration plot of *R*_ct_ versus iodate concentration

For this purpose, the modified electrode was introduced into the solutions containing various concentrations of iodate, and the results are also shown in Fig. 11a. It is obvious that the semicircle begins to change and its diameter decreased gradually with increasing iodate concentration. The calibration plot between 5 and 150 nM consists of two linear (5–50 and 70–150 nM) segments with different slopes, corresponding to two different ranges of iodate concentration (Fig. 11b).

In the concentration range of 5–50 nM, the correlation coefficient is 0.994 and the detection limit was calculated as 1.2 nM (based on signal/noise [*S*/*N*] = 3). Therefore, the proposed modified electrode can successfully detect iodate with high sensitivity and a low detection limit. The detection limit, linear calibration range, and applied potential for CySH and iodate detection are listed in Table 1. These analytical parameters for the proposed modified electrode are comparable or better than the results reported for CySH and iodate determinated at the surface of recently fabricated modified electrodes [33, 35–44].

**Table 1:** Electrochemical response of various modified electrodes for l-cysteine and iodate

Electrode	Analyte	Applied potential	LOD(µM)	Linear range (µM)	References
WO_3_/PANI/GCE	IO_3_^−^	−0.25	2.70	20.0–500	[35]
AuNPs/P-3MTP	IO_3_^−^	−0.29	1.40	5.00–500	[36]
SiNH_3_PW_12_-CPE	IO_3_^−^	−0.2	3.10	5.00–1000	[37]
MWCNTs/[C_8_Py][PF_6_]-PMo_12_/GCE	IO_3_^−^	0	15.0	20.0–2000	[38]
AuNPs/PEI/CNTs–COOH/ORC	IO_3_^−^	+0.15	0.17	1.00–2000	[39]
Pt/CNTs	CySH	+0.48	0.30	0.50–1000	[40]
Graphite/MWCNTs/AuNPs/CCE	CySH	+0.10	0.002	0.008–5.94	[41]
GNs/MnO_2_/GCE	CySH		0.108	1.44–1.73	[42]
Ordered mesoporous carbon	CySH	+0.47	0.10	3.00–130	[43]
MnO_2_–C/Chit/GCE	CySH		0.318	0.726–990	[44]
GC/AuNPs/PEI/GNs/ACA	IO_3_^-^	+0.11	0.002	0.005–1.30	[33]
	CySH	+0.05	0.30	1.00–15.0	
AgNPs/CNT–COOH/rGO/Mo	IO_3_^−^	+0.45	1.2 nM	5–400 nM	
	CySH	+0.05	300 pM	1.0–37.0 nM	

WO_3_/PANI, tungsten oxide/polyaniline; GCE, glassy carbon electrode; P-3MTP, poly (3-methylthiophene) composites; 3-SiNH_3_-PW_12_, α PW_12_O_40_^−3^ supported on the surface of aminopropyl(triethoxy)silane; CPE, carbon paste electrode; MWCNTs/[C_8_Py][PF_6_]-PMo_12_, multiwalled carbon nanotubes/n-octylpyridinum hexafluorophosphate and 1:12 phosphomolybdic acid; PEI, polyethyleneimine; ORC, organoruthenium(II) complexes; CCE, carbon ceramic electrode; GNs, graphene nanosheets; Chit, chitosan

### Study of Stability and Reproducibility

In order to evaluate the long-term stability of the modified electrode and the reversibility of electrochemical behavior, the current and potential response of AgNPs/CNT/rGO/MoO_2_/Sal-His modified electrode were recorded before and after storing the electrode in ambient condition for 1 week. The response of modified electrode after 1 week indicates that the current and potential response of sensor remain almost unchanged. In addition, the operational stability of the modified electrode was monitored by recording 100 repetitive cycles at a scan rate of 50 mV s^−1^. No detectable change was observed in the peak height and potential separation after 100 repetitive cycles. The high stability of AgNPs/CNT/rGO/MoO_2_/Sal-His modified electrode may be related to the mechanical and chemical stability of MoO_2_/Sal-His film at the surface of electrode, which can lead to its stabilization against desorption and avoid from leaching into the solution.

Furthermore, the reproducibility of the MoO_2_/Sal-His/AgNPs/CNT/rGO/GC modified electrode was tested by five repetitive measurements at various concentrations of CySH (5, 10, and 20 nM). The acceptable and reproducible signals with relative standard deviations (RSD) of 5.5, 6.1, and 4.1 % are obtained.

## Conclusion

In this study, a new bifunctional electrocatalyst was introduced and used as a modifier at the surface of GC electrode. The suitability of AgNPs/CNT/rGO/MoO_2_/Sal-His as an ideal catalyst toward iodate reduction and CySH oxidation was demonstrated. The proposed bifunctional modified electrode exhibits significant advantages such as low overpotential, high current density response, high sensitivity for iodate and CySH determination, and very good reproducibility and stability. It can be used as an impedimetric electrochemical sensor for monitoring of iodate and CySH.
